# Comparative Transcriptomic Analysis of *Gossypium hirsutum* Fiber Development in Mutant Materials (*xin w 139*) Provides New Insights into Cotton Fiber Development

**DOI:** 10.3390/plants13081127

**Published:** 2024-04-17

**Authors:** Chunping Li, Jieyin Zhao, Zhongshan Liu, Yanlong Yang, Chengxia Lai, Jun Ma, Alifu Aierxi

**Affiliations:** 1 Research Institute of Economic Crops, Xinjiang Academy of Agricultural Sciences, Urumqi 830091, China; chunpin96@126.com (C.L.); 13565893673@163.com (Z.L.); yangyl0629@163.com (Y.Y.); lchxia2001@163.com (C.L.); 2 Engineering Research Centre of Cotton, Ministry of Education/College of Agriculture, Xinjiang Agricultural University, 311 Nongda East Road, Urumqi 830052, China; cottonzjy@126.com

**Keywords:** *Gossypium hirsutum*, mutant, fiber development, RNA-seq, candidate genes

## Abstract

Cotton is the most widely planted fiber crop in the world, and improving cotton fiber quality has long been a research hotspot. The development of cotton fibers is a complex process that includes four consecutive and overlapping stages, and although many studies on cotton fiber development have been reported, most of the studies have been based on cultivars that are promoted in production or based on lines that are used in breeding. Here, we report a phenotypic evaluation of *Gossypium hirsutum* based on immature fiber mutant (*xin w 139*) and wild-type (Xin W 139) lines and a comparative transcriptomic study at seven time points during fiber development. The results of the two-year study showed that the fiber length, fiber strength, single-boll weight and lint percentage of *xin w 139* were significantly lower than those of Xin W 139, and there were no significant differences in the other traits. Principal component analysis (PCA) and cluster analysis of the RNA-sequencing (RNA-seq) data revealed that these seven time points could be clearly divided into three different groups corresponding to the initiation, elongation and secondary cell wall (SCW) synthesis stages of fiber development, and the differences in fiber development between the two lines were mainly due to developmental differences after twenty days post anthesis (DPA). Differential expression analysis revealed a total of 5131 unique differentially expressed genes (DEGs), including 290 transcription factors (TFs), between the 2 lines. These DEGs were divided into five clusters. Each cluster functional category was annotated based on the KEGG database, and different clusters could describe different stages of fiber development. In addition, we constructed a gene regulatory network by weighted correlation network analysis (WGCNA) and identified 15 key genes that determined the differences in fiber development between the 2 lines. We also screened seven candidate genes related to cotton fiber development through comparative sequence analysis and qRT–PCR; these genes included three TFs (*GH_A08G1821* (bHLH), *GH_D05G3074* (Dof), and *GH_D13G0161* (C3H)). These results provide a theoretical basis for obtaining an in-depth understanding of the molecular mechanism of cotton fiber development and provide new genetic resources for cotton fiber research.

## 1. Introduction

Cotton is a highly significant fiber crop that is extensively cultivated worldwide. Over the years, there has been a strong focus on enhancing the quality of cotton fibers, which has emerged as a prominent area of research. Cotton breeders have been actively engaged in developing and cultivating germplasm resources that not only yield high quantities but also possess exceptional fiber quality [1,2]. Cotton fiber development is a complex process that occurs in four consecutive and overlapping stages. These stages include fiber initiation, elongation, thickening of the secondary cell wall, and maturation. Fiber initiation occurs from three days before flowering to three days post anthesis (DPA). Elongation occurs from 3 to 16 DPA. During this period, the cotton fibers undergo significant elongation as they grow in length. The secondary cell wall (SCW) stage typically spans from 16 to 40 DPA. During this phase, the cotton fibers undergo SCW deposition, resulting in increased fiber diameter and strength. The thickening of the SCW is an important process in enhancing the overall quality and strength of cotton fibers. The final stage of cotton fiber development is maturation, which occurs from 40 to 50 DPA [3]. The number of fibroblast-differentiated protrusions affects the number of mature fibers to a certain extent, and the progression of rapid elongation through primary wall synthesis determines the length of the mature fibers. Cellulose is deposited in the SCW, and the cell wall is thickened during the thickening period of the SCW stage [4,5,6]. Scientists are working to elucidate the key regulatory mechanisms involved in fiber development, including fibroblast differentiation, cell wall synthesis, and cellulose biosynthesis [7,8,9]. Cotton fibers develop from ovule epidermal cells, and improving cotton fiber quality has long been a research hotspot. The creation and breeding of germplasm resources with high yields and excellent fiber quality has also become a long-term research direction and goal for cotton breeders [1,2]. Studying the regulatory mechanism of cotton fiber cell elongation and secondary wall development is highly important for improving the cotton yield and fiber quality.

In recent years, there has been significant progress in sequencing technologies, leading to their widespread use and continuous optimization. This has resulted in a growing body of evidence highlighting the crucial role of RNA transcriptional regulation in plant growth and development [10]. Transcriptome sequencing (RNA-seq), one of the most commonly used second-generation high-throughput sequencing methods, has been widely used in the study of cotton fibers [11,12]. Through transcriptomics, many key regulatory pathways and gene expression patterns associated with fiber development have been revealed [13,14,15]. These findings have helped identify the key genes that control fiber development and have provided important insights for improving cotton fibers. Through RNA-seq of 0–35 DPA fibers of PimaS-7 and 5917, 4 candidate genes related to fiber strength were identified [16]. RNA-seq analysis at different stages of fiber development (7, 14, and 26 DPA) in the Coker 312 cotton variety allowed the identification of transcription factors and functional genes associated with this process. These genes encode proteins involved in various functional and metabolic pathways, including those involved in catalytic activity, carbohydrate metabolism, cell membrane and organelle functions, and signal transduction [17]. Analysis of the RNA-seq data at 10 and 20 DPA from fibers from 4 wild cotton species and 5 domesticated cotton species showed that wild cotton plants allocate more resources to the stress response pathway and that acclimation may lead to a reprogramming of resource allocation in the direction of increasing fiber growth via regulation of the stress response network [18]. Through RNA-seq analysis of the offspring of the Xinhai 16 and landy cotton line 9 backcrossed populations at different stages of fiber development (0, 5, 10, and 15 DPA), researchers identified 21 genes closely associated with fiber development. These genes encode proteins involved in processes such as cell wall relaxation, microtubule formation, and the cytoskeletal structure of the cell wall [14]. Comparative transcriptomic analysis of TM-1, Hai7124 and 3-79 revealed that the expression at 3 DPA was the most different, and several candidate genes related mainly to SCW synthesis were identified based on WGCNA and time-course analysis [19].

Although RNA-seq has revealed many key regulatory networks and genes involved in cotton fiber development, providing important clues for improving cotton fiber quality, most related studies have been based on cultivars that are promoted in production or based on lines that are used in breeding. Natural fiber mutants are important germplasm resources for deciphering the development mechanism of long fibers and short fibers due to their unique characteristics. In the early 1970s, an immature fiber (*im*) mutant was discovered in the upland cotton variety Acala 4-42 [20]. Due to defects in the SCW development, this mutant has decreased fiber weight and fineness. Through RNA-seq analysis of *im* mutant fibers compared to those of the near-isogenic wild-type line (NIL) TM-1, it was found that the genes associated with cellulose synthesis, SCW biogenesis, cell wall thickening, and sucrose metabolism were significantly upregulated in TM-1 [21]. Additionally, the decreased fiber cell wall thickness in the *im* mutant fibers was found to be associated with the dysregulation of genes involved in the stress response and cellular respiration [21]. Through hybridization of *im* with five different strains, F_2_ and F_2:3_ populations were generated, further confirming the negative impact of the *im* mutant on fiber quality traits and the lint yield [22]. The analysis of multiple genetic factors revealed that a 22 bp deletion in the gene encoding the pentapeptide repeat (PPR) motif (imPPR, *Gh_A03G0489*) was associated with the fiber phenotype of the im mutant [23]. Based on the transcriptome data of Xuzhou142 and its fiber mutant *xu142 fl*, as well as three different lint-yield strains derived from the hybridization of these two lines at 0 DPA and 5 DPA, it was confirmed through virus-induced gene silencing (VIGS) experiments that two long noncoding RNAs (XLOC_545639 and XLOC_039050) can increase the number of ovule initials in *xu142 fl* [24]. Herein, we first identified and evaluated the 2-year field agronomic traits, yield traits and fiber quality of the *G. hirsutum* line Xin W139 and its fiber development mutants (*xin w 139*), and RNA-seq was carried out at 7 stages of fiber development. These results provide a theoretical basis for an in-depth understanding of the molecular mechanism of cotton fiber development and provide new genetic resources for cotton fiber research.

## 2. Results

### 2.1. Phenotypic Identification of Xin W 139 and xin w 139

**Figure 1:**
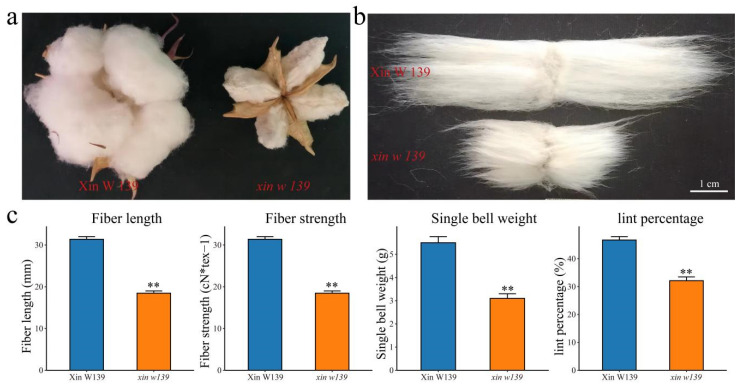
(**a**) Phenotypes of Xin W 139 and *xin w 139* after maturation. (**b**) Phenotype of the length of mature fibers of Xin W 139 and *xin w 139*. Bar = 1 cm. (**c**) Statistical analysis of the fiber length, fiber strength, single-boll weight and lint percentage of Xin W 139 and *xin w 139*. Significant differences were determined by a *t* test using a one-way ANOVA (** *p* < 0.01).

In 2022 and 2023, we identified and evaluated the agronomic traits, yield traits, and fiber quality traits of Xin W 139 and its fiber development mutant, *xin w 139*, respectively. The fiber length, fiber strength, single-boll weight and lint percentage of *xin w 139* were significantly lower than those of Xin W 139, but there were no significant differences in the plant height, fruit branch number or boll number per plant between the two lines (Figure 1c and Figure S1). This finding suggested that the mutant lines we found can be reliably used for further study. To further investigate the molecular mechanism and candidate genes associated with the differences in fiber development between Xin W 139 and *xin w 139*, RNA-seq was performed on samples from 7 developmental time points (0 DAP, 5 DAP, 10 DAP, 15 DAP, 20 DAP, 25 DAP and 30 DAP) for the fibers of the 2 lines.

### 2.2. RNA-Seq Analysis

**Figure 2:**
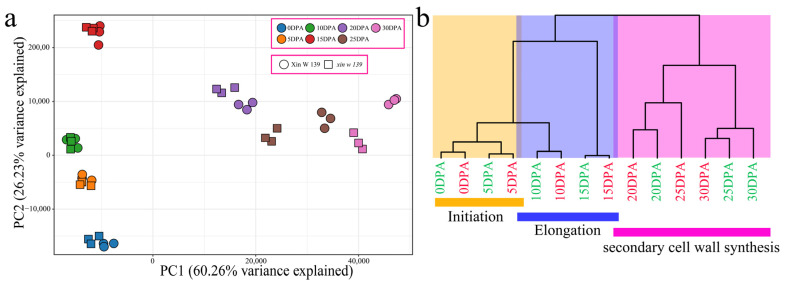
(**a**) PCA of 42 RNA-seq samples. (**b**) Cluster dendrogram showing three different developmental stages of cotton fibers: initiation, elongation and secondary wall synthesis (the green font represents Xin W 139, and the red font represents *xin w 139*).

A total of 42 samples from 2 lines and 7 fiber development stages were filtered by RNA-seq, and a total of 309.76 Gb of clean data were obtained. The minimum size of the clean data for each sample in this study was 6.64 Gb. The percentage of Q30 bases, which represents the quality of the sequencing reads, exceeded 92.32%. The GC content, an indicator of the proportion of guanine (G) and cytosine (C) bases in the DNA sequence, exceeded 43.56%. Furthermore, the alignment rate of the reads with the reference genome ranged from 97.04% to 98.45%, with an average alignment rate of 98.07% (Table S2). Principal component analysis (PCA) revealed that the transcriptome data were reliable and reproducible, as evidenced by the clustering of biological replicates (Figure 2a). To validate the accuracy of the transcriptome expression profile, six genes were randomly chosen for qRT–PCR analysis, which was performed with three independent replicates. Subsequently, the correlation between the qRT–PCR and RNA–seq data was calculated (Figure S2). The analysis demonstrated a significant correlation between the transcriptome data and the qRT–PCR data, confirming the reliability of the transcriptome sequencing results.

To further understand the transcriptome dynamics of cotton fiber development, we performed principal component analysis (PCA) and hierarchical clustering (Figure 2a,b) on samples from two lines and seven developmental time points. Consistent with previously reported results for upland cotton fiber development, our RNA-seq data could be divided into three groups, each corresponding to a specific fiber development stage (Figure 2b). The samples from the earliest time points (0~5 DPA) formed the first group, which represented the initiation of fiber development; the samples collected between 5 DPA and 15 DPA formed the second group, which represented the elongation of fibers; and the samples collected between 15 DPA and 30 DPA belonged to the third group, corresponding to the secondary wall synthesis of fibers. The initiation and elongation of fiber development were highly similar between the lines, indicating that their transcriptional regulatory patterns were highly similar. Interestingly, there were differences in the clustering of SCW synthesis between Xin W 139 and *xin w 139*. The clustering observed for the 25 DPA period of *xin w 139* was closer to that for Xin W 139 at 20 DPA, and the clustering observed for the 30 DPA period of *xin w 139* was more closely related to that for the 25 DPA period of Xin W 139. This shows that the main reason for the decrease in the fiber strength and length of *xin w 139* may be the difference in fiber development after 20 DPA.

### 2.3. Differential Expression Analysis within Lines

**Figure 3:**
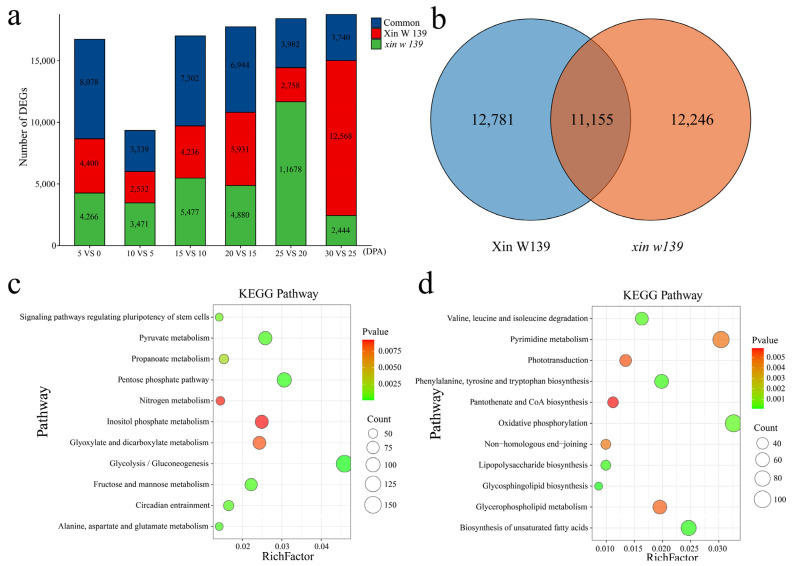
(**a**) Histogram showing the number of specific and common DEGs in the two lines. (**b**) Venn diagram of all the DEGs in Xin W 139 and *xin w 139*. (**c**) KEGG enrichment analysis of all the DEGs in Xin W 139. (**d**) KEGG enrichment analysis of all the DEGs in *xin w 139*.

To study the differential regulatory patterns of transcription at different stages of fiber development in the two lines, we identified DEGs at each stage of fiber development in the two lines (Figure 3a and Figure S3). Compared with those at 0 DPA, the number of DEGs common to the 2 lines at 5 DPA was 8078, accounting for 48.2%, with 4400 unique DEGs in Xin W 139 and 4266 unique DEGs in *xin w 139*. Compared with those at 5 DPA, the number of DEGs common to the 2 lines at 10 DPA was 3339, accounting for 35.7%, with 2532 unique DEGs in Xin W 139 and 3471 unique DEGs in *xin w 139*. Compared with those at 10 DPA, the number of DEGs shared by the 2 lines at 15 DPA was 7302, accounting for 42.9%, with 4236 unique DEGs in Xin W 139 and 5477 unique DEGs in *xin w 139*. Compared with those at 15 DPA, the number of DEGs common to the 2 lines at 20 DPA was 6944, accounting for 39.1%, with 5931 unique DEGs in Xin W 139 and 4880 unique DEGs in *xin w 139*. Compared with those at 20 DPA, there were 3982 DEGs (21.6%) at 25 DPA, 2758 in Xin W 139 and 11678 in *xin w 139*. Compared with those at 25 DPA, there were 3740 DEGs (19.9%) at 30 DPA, 12568 DEGs in Xin W 139 and 2444 DEGs in *xin w 139*. A total of 11,855 DEGs were recorded during the 2 different fiber development periods, with 12,781 DEGs in Xin W 139 and 12,246 DEGs in *xin w 139* (Figure 3b). Through enrichment analysis of the DEGs, it was found that the genes that were differentially expressed in only Xin W 139 were annotated mainly to pathways related to sugar and amino acid metabolism (Figure 3c). The genes that were differentially expressed in only the *xin w 139* subgroup were annotated mainly in the sugar, amino acid, biosynthesis of unsaturated fatty acid, and phosphorylation-related metabolic pathways (Figure 3d).

**Figure 4:**
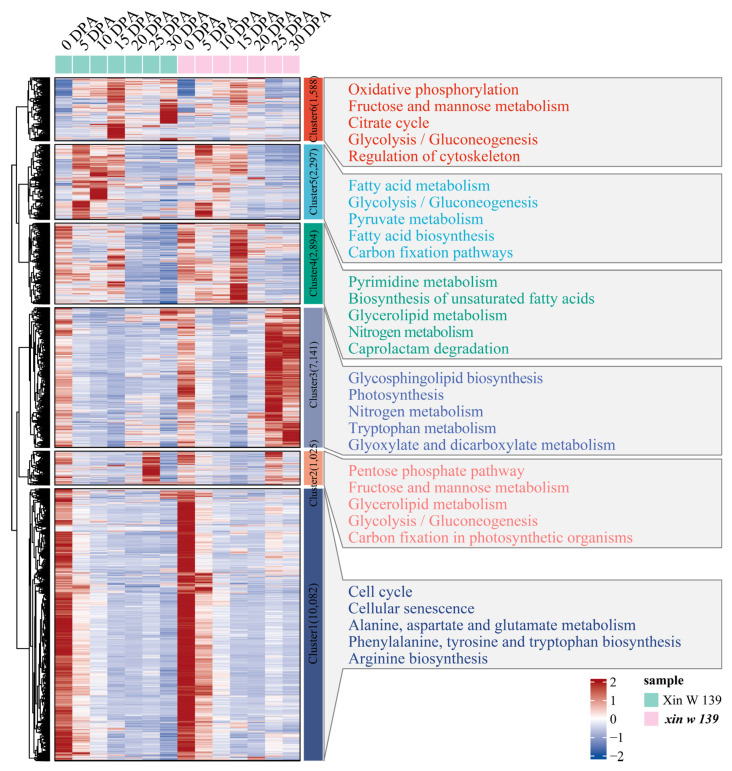
Expression pattern and functional enrichment analysis of DEGs in the lines. The right side shows the KEGG annotation results of each cluster, showing the top 5 pathways with the smallest *p* values.

To further understand the functional transformation of fiber development in the 2 lines, we clustered 25,027 genes that were specifically and differentially expressed between the 2 lines into 6 clusters using the k-means clustering algorithm and then performed KEGG annotation for each cluster functional category (Figure 4). Cluster 2 was highly expressed at 25 DPA in Xin W 139 and was mainly annotated to glucose metabolism and carbon fixation in the photosynthetic organism pathways. Cluster 3 was highly expressed at 25 DPA in *xin w 139* and was mainly annotated in the glycosphingolipid biosynthesis, photosynthesis, nitrogen metabolism, tryptophan metabolism and glyoxylate and dicarboxylate metabolism pathways. Cluster 4 was highly expressed at 15 DPA in *xin w 139* and was mainly annotated to the pyrimidine metabolism, biosynthesis of unsaturated fatty acids, glycerolipid metabolism, nitrogen metabolism and caprolactam degradation pathways. Cluster 5 was highly expressed at 15 DPA and 30 DPA in Xin W 139 and was mainly annotated to the oxidative phosphorylation, fructose and mannose metabolism, citrate cycle, glycolysis/gluconeogenesis and regulation of the cytoskeleton pathways.

### 2.4. Analysis of Differential Expression between Lines

**Figure 5:**
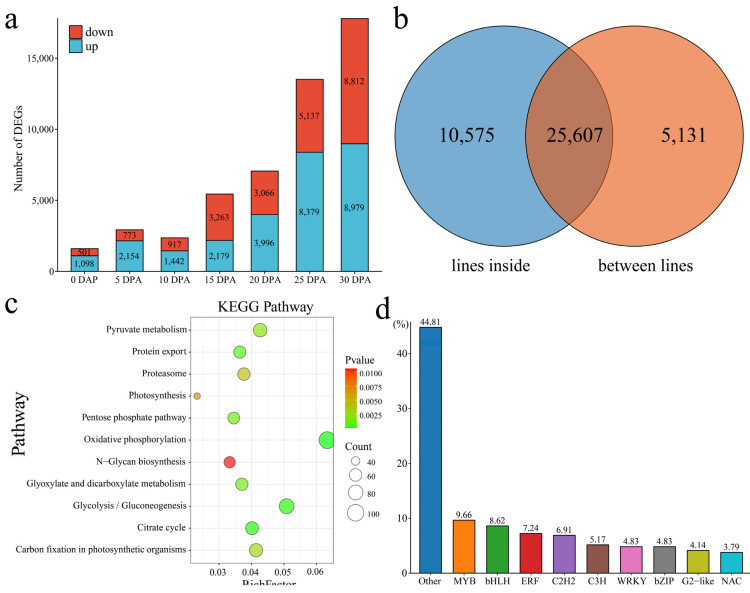
(**a**) Number of DEGs at different stages of fiber development between the lines. (**b**) Venn diagram of the number of DEGs between and inside the lines; the lines inside represent the DEGs between the same line and different DPAs, and the lines between lines represent the DEGs between the same DPAs of Xin W 139 and *xin w 139*. (**c**) Line-specific KEGG enrichment analysis of the DEGs. (**d**) Histogram of the percentage of the line-specific DEGs among the TFs.

We identified the DEGs of Xin W 139 and *xin w 139* at each stage of upland cotton fiber development (Figure 5a). There were a total of 30,738 DEGs, with 1098 upregulated and 501 downregulated at 0 DPA, 2154 upregulated and 773 downregulated at 5 DPA, 1442 upregulated and 917 downregulated at 10 DPA, 2179 upregulated and 3263 downregulated at 15 DPA, 3996 upregulated and 3006 downregulated at 20 DPA, 8379 upregulated and 5137 downregulated at 25 DPA, and 8979 upregulated and 8812 downregulated at 30 DPA. With the development of fibers, the number of DEGs between the 2 lines also increased, peaking at 30 DPA (Figure 5a). Among the 36,182 DEGs at different developmental stages within the lines, 25,607 common DEGs and 5131 DEGs were unique to the 2 lines (Figure 5b). According to the enrichment analysis, the 5131 DEGs were mainly annotated to the signaling pathways related to glucose metabolism, photosynthesis, oxidative phosphorylation and the citrate cycle (Figure 5c). A total of 290 transcription factors, mainly MYB, bHLH, ERF, and C2H2, were identified among the 5131 DEGs (Figure 5d). The immature phenotype was mainly regulated by the pentatricopeptide repeat (PPR) protein [23]. We identified 523 genes encoding PPR proteins among the DEGs, and the expression levels at 0 DPA and 5 DPA were the highest in the 2 lines (Table S3).

**Figure 6:**
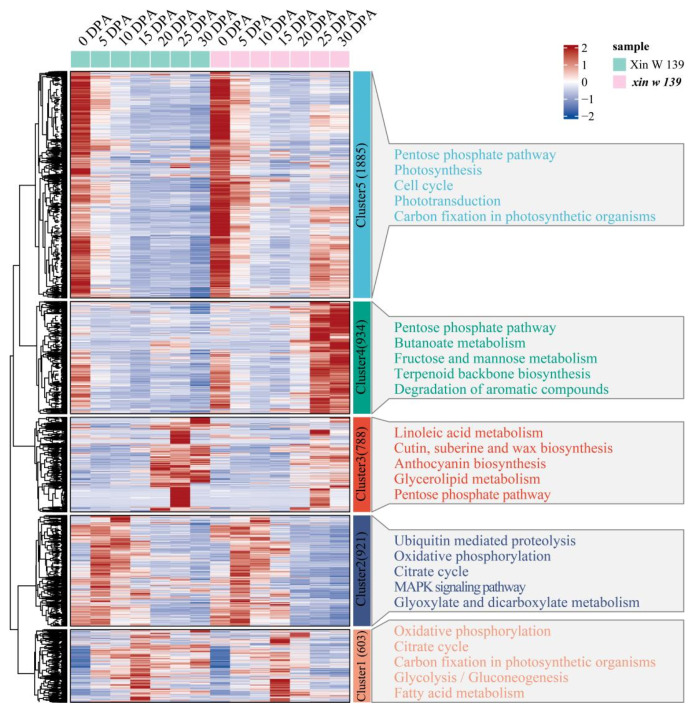
Specific expression of DEGs between lines, expression patterns and functional enrichment analysis. The right side shows the KEGG annotation results of each cluster, showing the top 5 pathways with the smallest *p* values.

We clustered 5131 genes that were specifically and differentially expressed between the 2 lines into 5 clusters using the k-means clustering algorithm and then performed KEGG annotation for each cluster functional category (Figure 6). Cluster 3 was expressed specifically at 20 DPA, 25 DPA and 30 DPA in Xin W 139, and the main annotations were linoleic acid metabolism; cutin, suberin and wax biosynthesis; anthocyanin biosynthesis; glycerolipid metabolism; and the pentose phosphate pathway. Cluster 4 was highly expressed at 25 DPA in *xin w 139*, and the main annotations were the pentose phosphate pathway, butanoate metabolism, fructose and mannose metabolism, terpenoid backbone biosynthesis, and the degradation of aromatic compounds pathway. Cluster 5 was highly expressed at 25 DPA and 30 DPA in *xin w 139* and was mainly annotated to the pentose phosphate pathway, photosynthesis, the cell cycle, phototransduction, and carbon fixation in the photosynthetic organism pathway.

### 2.5. WGCNA

**Figure 7:**
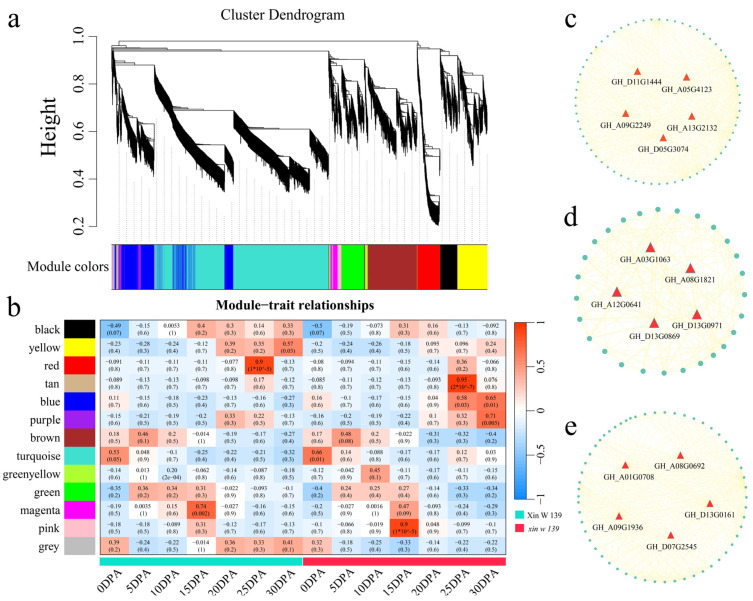
(**a**) Hierarchical clustering tree of genes based on the co-expression network analysis. (**b**) Heatmap of correlations and significance between the modules and different periods of fiber development. (**c**) Gene co-expression network within the red module. (**d**) Gene co-expression network within the tan module. (**e**) Gene co-expression network within the pink module.

To study the gene regulatory network involved in cotton fiber development, we used weighted gene co-expression network analysis (WGCNA) to construct a co-expression network for 5131 DEGs in the lines (the β soft threshold was 9, and the no scale R^2^ > 0.8), and a total of 13 co-expression modules (Figure 7a and Figure S4) were obtained. According to the analysis of the correlation between the modules and lines, the red module was significantly highly correlated with Xin W 139 at 25 DPA (r = 0.90, *p* < 0.01), the tan module was significantly highly correlated with *xin w 139* at 25 DPA (r = 0.95, *p* < 0.01), and the pink module was significantly highly correlated with *xin w 139* at 15 DPA (r = 0.90, *p* < 0.01) (Figure 7b). For each module, the 5 genes with the highest degree of linkage were identified as hub genes, and 15 hub genes were ultimately identified (Figure 7c–e). Among the 15 genes, 2 encoded bHLH transcription factors (*GH_A08G1821* and *GH_D11G1444*), 1 encoded a WRKY transcription factor (*GH_D07G2545*), 1 encoded a Dof transcription factor (*GH_D05G3074*), 1 encoded an SBP transcription factor (*GH_D13G0869*) and 1 encoded a C3H transcription factor (*GH_D13G0161*).

### 2.6. Identification of Candidate Genes

**Figure 8:**
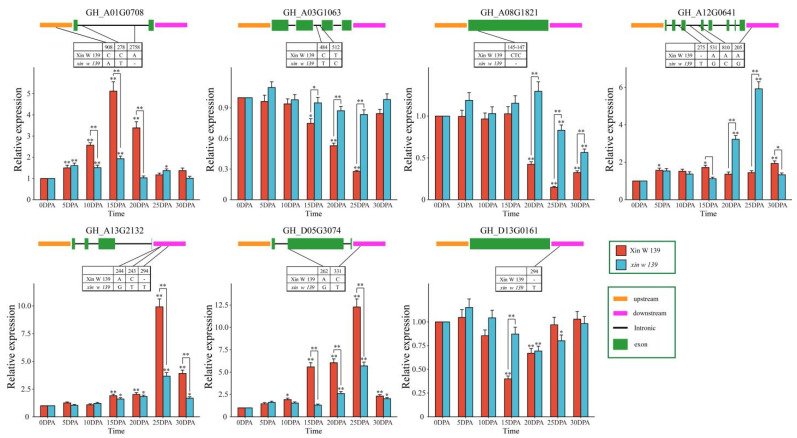
SNP/Indel information and expression profiling of 7 candidate genes between Xin W 139 and *xin w 139*. The results are presented as the means ± SDs (n = 3, * *p* < 0.05, ** *p* < 0.01).

To further analyze the relationship between these 15 hub genes and cotton fiber development, sequence comparison analysis between the 2 lines (Xin W 139 and *xin w 139*) was carried out based on RNA-seq data, and 7 genes (*GH_A01G0708*, *GH_A03G1063*, *GH_A08G1821*, *GH_A12G0641*, *GH_A13G2132*, *GH_D05G3074* and *GH_D13G0161*) were found to contain SNPs/Indels in the upstream, downstream, exon, or intron regions, with *GH_A08G1821* (bHLH), *GH_D05G3074* (Dof) and *GH_D13G0161* (C3H) being transcription factors (Figure 8). Then, qRT–PCR was used to determine that the expression patterns of *GH_A01G0708*, *GH_A13G2132*, and *GH_D05G3074* increased significantly with the development of fibers, and the expression levels in Xin W 139 were significantly greater than those in *xin w 139*. The expression levels of three genes (*GH_A03G1063*, *GH_A08G1821* and *GH_D13G0161*) decreased significantly in Xin W 139 with fiber development, decreased slightly in *xin w 139* and were significantly greater than those in Xin W 139. The expression level of *GH_A12G0641* in Xin W 139 increased slightly with fiber development, and the expression level in *xin w 139* increased significantly and was greater than that in Xin W 139. In conclusion, we screened seven candidate genes related to cotton fiber development, including three TFs (bHLH, Dof and C3H), via sequence comparison analysis and qRT–PCR.

## 3. Discussion

Cotton is a widely planted natural fiber crop worldwide, and improving cotton fiber quality has long been a research hotspot. The creation and breeding of germplasm resources with high yields and excellent fiber quality has also become a long-term research direction and goal for cotton breeders [1,2]. Cotton fibers develop from single cells of the ovule epidermis, and their development involves a complex process consisting of four consecutive but overlapping stages. Most related studies have been based on cultivars that are promoted in production or based on lines that are used in breeding; especially, there are few reports on natural mutants [25,26,27]. Here, we performed phenotypic evaluation and comparative transcriptomic studies at seven time points during cotton fiber development in immature fiber mutant (*xin w 139*) and wild-type (Xin W 139) lines. The fiber length, fiber strength, single-boll weight and lint percentage of *xin w 139* were significantly lower than those of Xin W 139, and there were no significant differences in the other traits (Figure 1 and Figure S1). PCA and cluster analysis of the RNA-seq data revealed that these time courses could be clearly divided into three distinct groups corresponding to the initiation, elongation, and secondary wall synthesis stages of fiber development. We detected a decrease in the fiber length, strength and single-boll weight of *xin w 139*, mainly due to its development after 20 DPA. The 20–40 DPA period mainly encompasses the secondary wall synthesis stage of cotton fiber development, during which a large amount of cellulose is synthesized, making this the key period for improving fiber strength [1]. We found that the fiber length of *xin w 139* was also reduced by approximately 40% compared to that of Xin W 139, indicating that fiber elongation was not completed at 20 DPA. At 20 DPA, the transition from the fiber elongation stage to the fiber secondary wall synthesis stage occurs [28]. At 20 DPA, the SCW begins to be deposited on the inner side of the primary wall so that the elongation of the fibroblasts and the thickening of the SCW overlap for a period [29]. Cellulose is twisted in the cell wall along the direction of the long chain, several cellulose molecules twist together to form cellulose microfibrils, and 90% of the cellulose in mature cotton fibers is deposited during this period [30]. At this stage, a unique winding cell wall layer is formed, similar to the S1 layer in wood fibers, and the fiber strength can be mainly attributed to the combined action of this cell wall layer and secondary wall cellulose deposition [30,31]. The results showed that 20 DPA was highly important for the development of cotton fibers and contributed greatly to the fiber length and strength. This provides important theoretical support for improving the length and strength of cotton fibers.

During the development of cotton fibers, cellulose is the most important product during the thickening stage of the SCW. Cellulose synthase (CesA) synthesizes a large amount of cellulose on the plasma membrane, and CesA gene expression increases at the same time [32]. There are 32 CesA genes in *G. hirsutum*, among which *GhCesA4*, *GhCesA7* and *GhCesA8* have high expression levels during the SCW stage, indicating that these 3 genes may be related to the development of the fiber SCW stage [33,34,35]. We also found that the expression of *GhCesA4*, *GhCesA7* and *GhCesA8* increased significantly during the secondary wall thickening stage, but there was no significant difference in the expression of these genes between *xin w 139* and Xin W 139, which indicates that the reason for the difference between these two lines is not related to the CesA gene. Sucrose synthase can directly convert the carbon in sucrose into cellulose or callose synthase in the plasma membrane. The appearance of the callose content peak may be an important sign that the secondary wall of the fiber cells has begun to thicken [36]. For all the DEGs we identified, clusters that were highly expressed during the SCW period were significantly enriched in sugar metabolism, the pentose phosphate pathway, butanoate metabolism, and the fructose and mannose metabolism signaling pathways. Previous studies on the genetic mapping of the *im* mutant revealed that the gene encoding the PPR protein (*Gh_A03G0489*) is related to the immature fiber phenotype of the *im* mutant [37]. Although the expression levels of *Gh_A03G0489* were significantly different between TM-1 and IM during the SCW period, the expression levels were relatively low [37]. The PPR gene family is the largest gene family identified to date [38]. They mainly act on mitochondria or chloroplasts to regulate organelle genes at the post-transcriptional level, thereby affecting plant growth and development [38]. We identified 523 genes encoding PPR proteins among the DEGs, and the expression levels in both lines were generally low (FPKM < 2). Notably, we found that two genes encoding PPR proteins (*GH_D11G0868* and *GH_A05G0653*) exhibited a greater than four-fold change in expression during the SCW period (Table S3). Mei et al. reported that *GH_A05G0653* was expressed mainly in the A subgenome of 4 *G. hirsutum* varieties at 20 DPA but showed no difference among the 3 *G. barbadense* varieties [39]. We also found that the expression of *GH_A05G0653* in *xin w 139* at 20 DPA was greater than that in Xin W 139, which indicates that *GH_A05G0653* may be a negative regulator of fiber quality. The specific function and mechanism require further study.

We also detected 5131 DEGs in the interline space, including 290 DEGs encoding TFs, which will undoubtedly be the subject of future functional genomics studies. TFs can bind to specific cis-acting elements in the promoter region of genes to promote or inhibit the transcription of specific genes [40]. The MYB–bHLH–WD40 (MBW) protein complex is widely involved in the development of plant trichomes, and current research on cotton fibers has mainly focused on this topic [1]. For example, *GhDEL65* (bHLH) regulates its expression during fiber development by binding the transcript product to the promoter element of the *GhMYB2* gene, similar to the role of *GL3* in *A. thaliana* epidermal hair development [41]. As one of the indispensable members of the MBW protein complex, the WD40 protein plays an important role in regulating the development and differentiation of plant epidermal cells [42]. The gene homologs of *GhTTG1* and *GhTTG3* of the Arabidopsis WD40 protein (TTG1)-encoding gene in cotton can restore the *A. thaliana* hairless phenotype to the wild-type phenotype [43,44]. MBW, composed of the GL1–GL3–TTG1 ternary protein complex, regulates the growth and development of *A. thaliana* epidermal hair by activating the expression of the *GL2* gene (HD-ZIP) downstream. The *GL2* homolog gene encoding the GaHOX1 protein was specifically expressed upon the initiation of cotton fiber differentiation, and the expression of the *GaHOX1* gene driven by the *GL2* promoter was found to restore the normal development of epidermal hair in mutants with a hairless phenotype [45]. The 290 TFs we detected mainly included MYB and bHLH TFs. Furthermore, seven key candidate genes related to cotton fiber development, including three TFs (bHLH, Dof and C3H), were screened by WGCNA, comparative sequence analysis and qRT–PCR. *GH_A08G1821* (bHLH) may be a negative regulator of fiber development, as with the development of cotton fibers, its expression was significantly downregulated in Xin W 139, while its expression in the mutant lines was almost unchanged. The genes also included the less studied Dof (*GH_D05G3074*) and C3H (*GH_D13G0161*) transcription factors. With the development of cotton fibers, *GH_D05G3074* expression was upregulated in both lines, but its expression in Xin W 139 was significantly greater than that in the mutant lines, and we speculated that this gene may be a positive regulator of fiber development. However, the expression level of *GH_D13G0161* decreased significantly, and the expression in the mutant lines was significantly greater than that in Xin W 139. However, the exact role of these genes and markers in *G. hirsutum* fiber development has yet to be determined. Furthermore, candidate markers (SNPs/Indel) were developed and Kompetitive Allele-Specific PCR (KASP) markers were verified through the fiber phenotypic data of the natural population, and the genes corresponding to the markers that can be accurately classified (fiber strength or length) were studied as key candidate genes [46]. Functional verification of the selected genes was carried out through the upland cotton CRISPR-Cas9-mediated gene knockout and genetic transformation technology system. At the same time, multiomics sequencing combined with molecular biology methods was used to reveal the mechanism of action and genetic rules of the candidate genes, providing theoretical support for further optimizing cotton breeding strategies. Carrying out genome-wide selection and molecular marker-assisted selection breeding for markers that can be accurately typed will not only speed up the selection of new cotton varieties but also improve the breeding efficiency.

The 5131 DEGs between the lines were divided into 5 clusters, and the functional categories of each cluster were annotated based on the KEGG database. Cluster 3 was highly expressed at 25 DPA in Xin W 139. Cluster 4 was specifically high at 25 DPA in *xin w 139*, and by annotating these two clusters, we found an overlapping signaling pathway, the pentose phosphate pathway. The pentose phosphate pathway produces NADPH, and NADPH is involved in fatty acid synthesis [47]. The expression of the ethylene synthesis gene *GhACO1* was upregulated after the addition of very long-chain fatty acids (VLCFAs) in vitro, which increased the ethylene content in cotton fibers, thereby promoting the elongation of cotton fibers [48]. A recent study showed that BRs regulate the synthesis of *GhKCS*-mediated VLCFAs through *GhBES1*, promoting fiber elongation [49]. We found that the DEGs in *xin w 139* were annotated to the biosynthesis of the unsaturated fatty acids pathway, suggesting that the difference in fiber length between the two lines may be regulated by the expression of genes involved in the metabolic pathways of unsaturated fatty acids. VLCFAs, via enhanced UDP-l-rhamnose and UDP-d-galacturonic acid biosynthesis, can also increase the elongation of cotton fibers, which may also regulate fiber elongation through the sphingolipid biosynthesis pathway [1]. However, the contribution of VLCFAs to fiber strength has not been reported. Due to the significant difference in fiber length and fiber strength between the two lines, we suspect that the pentose phosphate pathway contributes to both the lightness and length of the fibers; this hypothesis still needs to be verified, but our results can provide important information for subsequent studies.

## 4. Materials and Methods

### 4.1. Plant Materials

In this study, a fiber development mutant was found for the line Xin W 139, which was selected for breeding at the Institute of Economic Crops of Xinjiang Academy of Agricultural Sciences in 2018 and named *xin w 139* (see Figure 1a,b for the phenotype and fiber analysis of Xin W 139 and *xin w 139* after flocculation in 2022). The agronomic traits (plant height and number of fruiting branches), yield traits (number of bolls per plant, weight per boll and coating) and fiber quality traits (fiber length and fiber strength) of the Xin W 139 and *xin w 139* lines were determined in Manas County, Changji city, Xinjiang, in 2022 and in Toutai township, Wusu city, Xinjiang, in 2023. For the field experiment, 1 row of each cultivar was planted; each plot was 5 m long, the row spacing was 0.35 m, the plant spacing was 0.10 m, and the management measures were the same as those used in the local conventional field. The cotton bolls on the day of flowering were labeled 0 DPA and they were sampled at 11 a.m. at 0, 5, 10, 15, 20, 25 and 30 DPA. During the sampling, the cotton husk was quickly peeled off within 1 min of peaching, and the fibers were removed with tweezers and immediately placed in liquid nitrogen for cryopreservation (6 replicates of each sample, 3 for RNA-seq and 3 for qRT–PCR).

### 4.2. RNA Extraction, cDNA Library Preparation, and Sequencing

RNA extraction was performed using the TRIzol method, and the quality of the extracted RNA was assessed via 1% agarose gel electrophoresis [50]. The extracted total RNA was subsequently transported to the Maiwei Metabolism Company (Wuhan, China) on dry ice for sequencing, after which the extracted RNA was fragmented using a PCR plate with a magnetic plate holder. Reverse transcription of the fragmented mRNA to cDNA was performed using Superscript II and random primers (Invitrogen, Carlsbad, CA, USA). The RNA-seq library preparations were sequenced on an Illumina (San Diego, CA, USA) HiSeq 2500/X platform, and 150 bp paired-end reads were generated. The library fragments were purified with an AMPure XP system (Beckman Coulter, Beverly, MA, USA). Then, 3 µL of USER Enzyme (NEB, Ipswich, MA, USA) was incubated with size-selected, adaptor-ligated cDNA at 37 °C for 15 min, followed by 5 min at 95 °C, before PCR. Then, PCR was performed with Phusion High-Fidelity DNA polymerase, universal PCR primers and Index (X) Primer. Finally, the PCR products were purified (AMPure XP system), and the library quality was assessed on an Agilent Bioanalyzer 2100 system. Fastp software (version 0.23.4) was used to remove the adapter sequences and filter out low-mass reads and reads with more than 5% poly-N sequences to obtain clean reads that could be used for the subsequent analysis [51]. The genome (https://www.cottongen.org/species/Gossypium_hirsutum/ZJU-AD1_v2.1, accessed on 3 January 2024) of upland cotton TM-1 was used as a reference, HISAT2 was used for the read alignment, and String Tie was used to quantify the reads in the alignment [52,53].

### 4.3. Analysis of DEGs

The fragments per kilobase of exon per million fragments mapped (FPKM) is a measure that quantifies gene expression levels. The number of reads per million that aligned to exonic regions was calculated and normalized by the length of the exonic regions and the total number of mapped reads. The gene expression levels in this study were determined using the FPKM method. The fold change in gene expression was calculated using EdgeR 4.0 software based on the number of clean reads obtained from the gene alignment [54]. An FDR ≦ 0.01 and a |log2-fold change| ≧ 1 were used as the standards for screening differentially expressed genes (DEGs) [55]. KEGG is a database resource for understanding high-level functions and utilities of biological systems, such as cells, organisms and ecosystems, from molecular-level information, especially large-scale molecular datasets generated by genome sequencing and other high-throughput experimental technologies (http://www.genome.jp/kegg/, accessed on 5 January 2024). We used KOBAS software 3.0.3 to test the statistical enrichment of differentially expressed genes in the KEGG pathways. The DEG sequences were submitted to PlantTFDB (http://planttfdb.cbi.pku.edu.cn/, accessed on 5 January 2024) for TF prediction.

### 4.4. Construction of Co-Expression Networks

The expression profile of the DEGs was determined by dynamic branching cleavage using the R language WGCNA package; the weighting coefficient was close to 0.8 β, the correlation coefficient requirement was met, and β = 9 was selected as the weighting coefficient in this study [56]. The automatic network builder Blockwise Modules was used to construct the network to obtain gene co-expression modules. The number of genes contained in each module was unequal, and the modules with a similarity of 0.75 were combined with minModuleSize = 30 and Merge Cut Height = 0.25 as the standards. The correlation coefficient between the characteristic vector module eigengene (ME) of the module and the different time points of fiber development of the two lines was calculated. Visualization of the co-expression networks was performed using Cytoscape (version 3.10.1) software [57].

### 4.5. SNP/Indel Analysis

Based on the HISAT2 alignment of the reads of each sample to the reference genome sequence, the SNPs/indels were identified using GATK (version 3.2-2) software [58]. The GATK identification criteria were as follows: (1) no more than 3 consecutive single-base mismatches were present in the 35 bp range and (2) the SNP quality values that were standardized by a sequence depth greater than 2.0. SNP/Indel loci were annotated using SnpEff software (version 3.6) to annotate regions of the genome (gene upstream, downstream, exon, or intron regions) based on the location of the variant locus on the reference genome as well as the gene location information on the reference genome [59].

### 4.6. qRT–PCR

The total RNA was extracted using the RNAprep Pure Polysaccharide Polyphenol Plant Total RNA Isolation Kit from Tiangen (Beijing, China). The concentration of each RNA sample was measured using a NanoDrop 2000 spectrophotometer from Thermo Fisher Scientific (Waltham, MA, USA). cDNA was synthesized through reverse transcription of the RNA using the M-MLV RTase cDNA Synthesis Kit from TaKaRa (Kyoto, Japan). qRT–PCR analysis was conducted on a Bio-Rad CFX96 real-time PCR system from Mannheim Roche Diagnostics GmbH (Mannheim, Germany), with iTaq Universal SYBR Green Supermix from Takara Bio, Inc., in a reaction volume of 20 μL. For the qRT–PCR analysis, the following reaction program was used: an initial pre-denaturation step at 95 °C for 30 s, followed by 40 cycles of denaturation at 95 °C for 5 s, annealing at 60 °C for 5 s, and extension at 72 °C for 34 s. The relative quantitative analysis of the qRT–PCR results was performed using the 2^−ΔΔCt^ method [60]. The Ct value of the internal reference gene was subtracted from the Ct value of the target gene to obtain the ΔCt. The mean ΔCt value of the control group (0 DPA) was subtracted from each ΔCt of the treatment group to obtain the ΔΔCt. The final expression level was calculated via the 2^−ΔΔCt^ method. The internal reference gene used was *GhUBQ7*, and each experiment was conducted with three biological replicates. All the primers utilized in this study can be found in Table S1.

## 5. Conclusions

In conclusion, we used RNA-seq data from seven time points for natural mutant and wild-type lines to provide a reliable dataset for studying cotton fiber development. We not only identified 20 DPA as the key period for FS and length development but also identified several important regulatory pathways involved in fiber development by identifying the DEGs and TFs between lines. Cluster analysis was performed to divide the DEGs into clusters that could describe different stages of fiber development. In addition, seven candidate genes related to cotton fiber development, including three TFs, were screened by WGCNA, sequence comparative analysis and qRT–PCR. However, the exact role of these genes and markers in upland cotton fiber development has yet to be determined. Our results provide a theoretical basis for obtaining an in-depth understanding of the molecular mechanism of cotton fiber development and provide new genetic resources for cotton fiber research.

## Data Availability

The genome databases used were downloaded from COTTON GEN (https://www.cottongen.org/species/Gossypium_hirsutum/ZJU-AD1_v2.1, accessed on 3 January 2024). The RNA-seq data presented in the study are deposited in the NCBI repository under accession number PRJNA1063919.

## Supplementary Materials

The following supporting information can be downloaded at https://www.mdpi.com/article/10.3390/plants13081127/s1, Figure S1. Statistical analysis of the plant height, number of fruiting branches and number of bolls of the Xin W 139 and *xin w 139* plants revealed significant differences via a one-way ANOVA (*t* tests); Figure S2. Scatter plot of the correlation between the RNA-seq and qRT–PCR data; Figure S3. Histogram of the percentage of DEGs within the lines; Figure S4. The scale-free topology fit index was calculated as a function of the soft-thresholding power; the red line indicates that the R^2^ value was 0.8; Table S1. All the primers used in this study; Table S2. Summary statistics for the RNA-seq analysis of cotton fiber samples from *G. hirsutum*. Table S3. Expression levels of the 523 genes encoding PPR proteins among the DEGs.
