# Supplementary figures and images for: Comparative Transcriptomic Analysis of Gossypium hirsutum Fiber Development in Mutant Materials (xin w 139) Provides New Insights into Cotton Fiber Development
Source: Plants (Basel). 2024 Apr 17;13(8):1127. doi: 10.3390/plants13081127 (PMC11054599; doi:10.3390/plants13081127)

Plant height

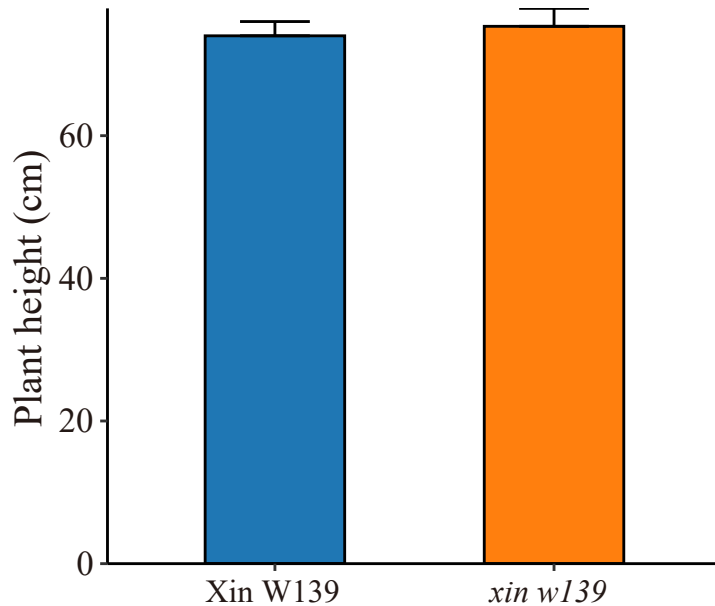

Number of fruit branches

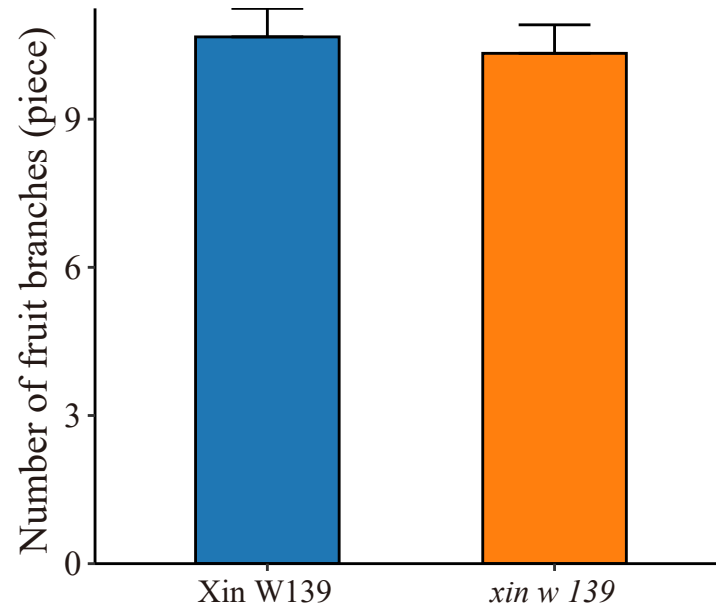

number of bells

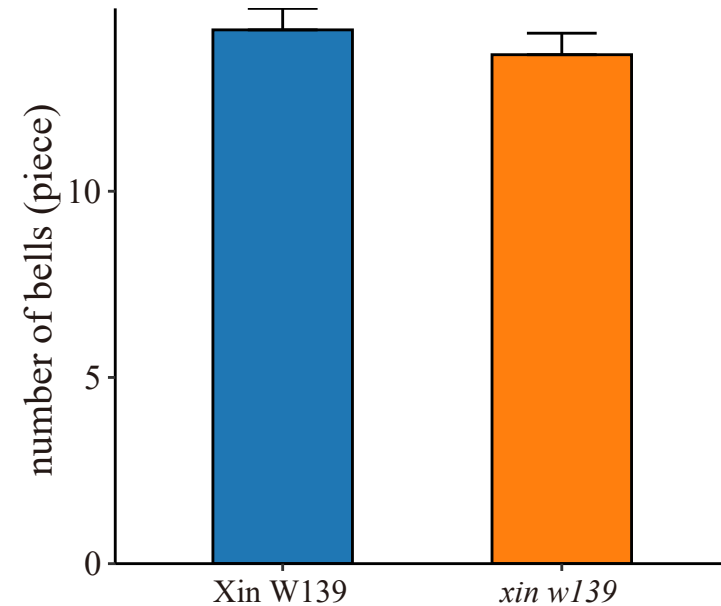

Supplement: Supplementary file 1 [file plants-13-01127-s001.zip › Fig S1.pdf]

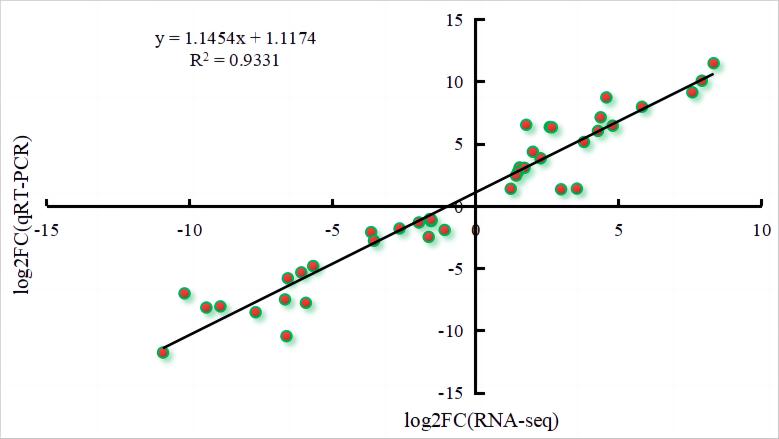

Supplement: Supplementary file 1 [file plants-13-01127-s001.zip › Fig S2.jpg]

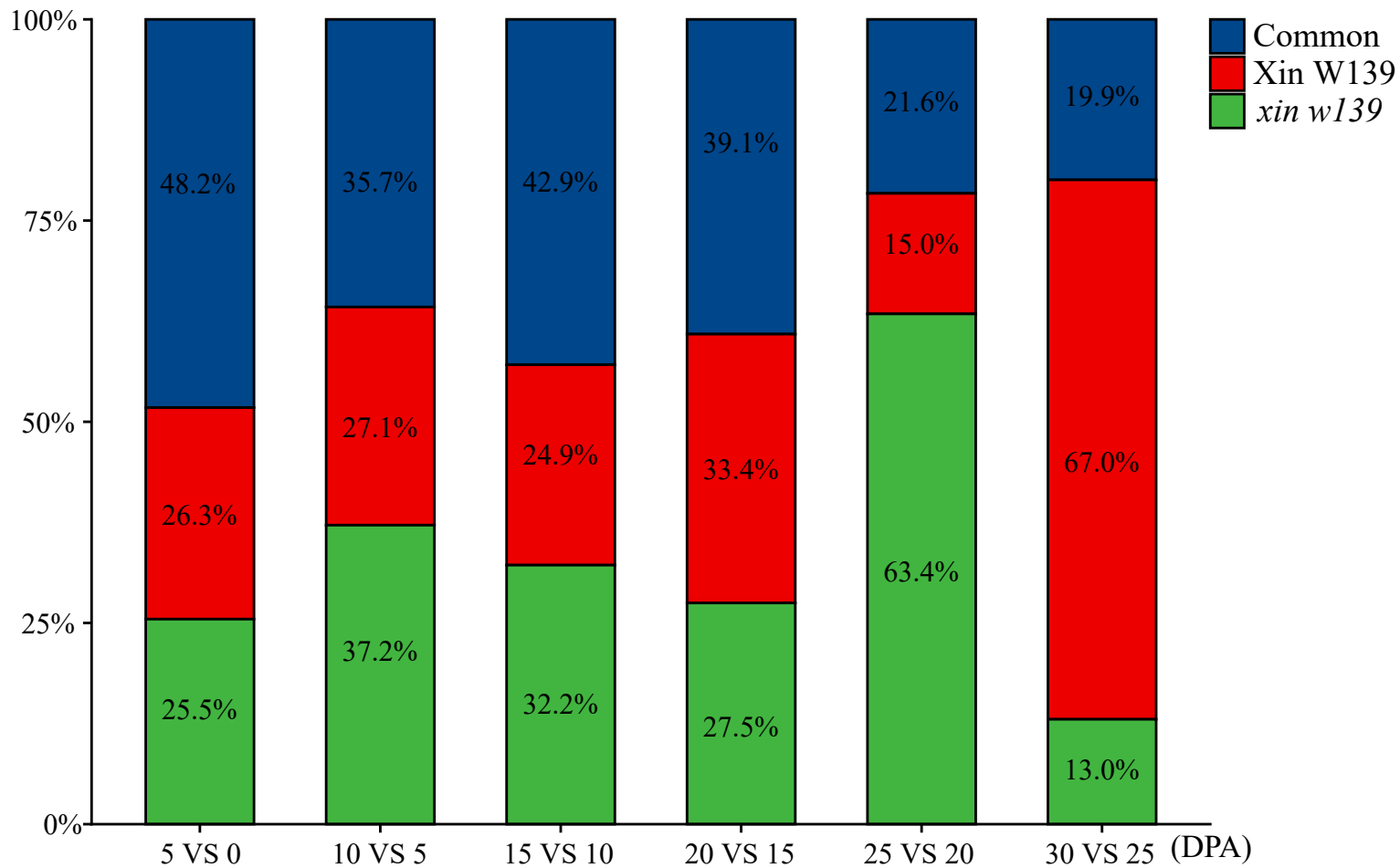

Supplement: Supplementary file 1 [file plants-13-01127-s001.zip › Fig S3.pdf]

Scale Free Topology Model Fit, signed  $R^2$

Scale independence

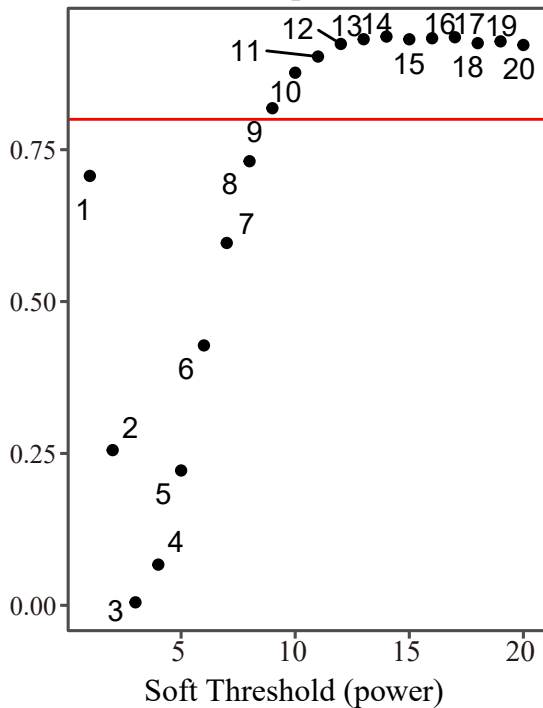

Mean connectivity

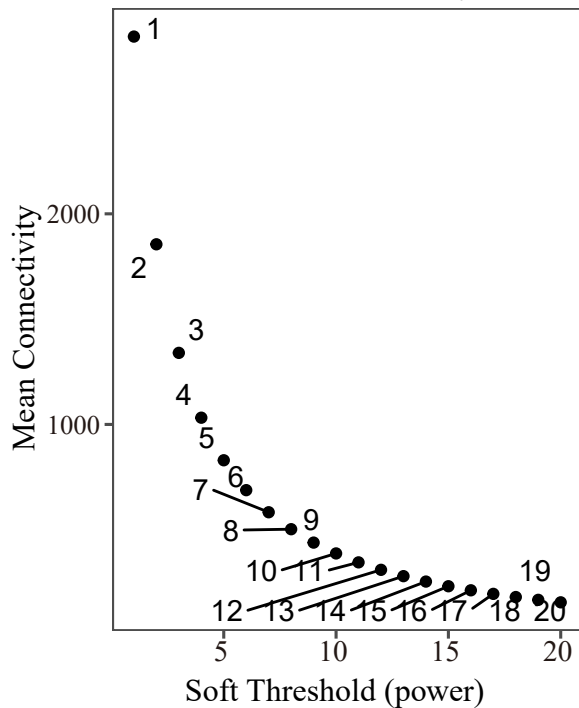

Supplement: Supplementary file 1 [file plants-13-01127-s001.zip › Fig S4.pdf]
